# Effectiveness and tolerability of low dose clindamycin for treatment of prosthetic joint infections, a retrospective cohort study

**DOI:** 10.1186/s12879-026-14378-0

**Published:** 2026-09-12

**Authors:** Jesper Ericsson, Tove Gustafsson, Olof Sköldenberg, Magnus Hedenstierna, Agata Rysinska, Johan Ursing

**Affiliations:** 1 Department of Clinical Sciences, Karolinska Institute, Danderyd Hospital, Entrevägen 2, Danderyd, Stockholm, 182 88 Sweden; 2 Department of Infectious Diseases, Danderyd Hospital, Entrevägen 2, Danderyd, Stockholm, 182 88 Sweden; 3 Department of Orthopaedics, Danderyd Hospital, Stockholm, Sweden

**Keywords:** Prosthetic joint infection, Effectiveness, Treatment outcome, Tolerability, Clindamycin, Rifampicin, *Staphylococcus* spp., *Streptococcus* spp., *Cutibacterium acnes*

## Abstract

**Background:**

Clindamycin is a potential drug for treating prosthetic joint infections, but pharmacokinetic monitoring shows that coadministration with rifampicin significantly reduces clindamycin concentrations. This study aimed to describe the real-world effectiveness and tolerability of clindamycin given alone or in combination with rifampicin or other antibiotics for treatment of PJI.

**Methods:**

This single-centre, retrospective cohort study, included patients treated for PJI from 2010 to 2022. PJI was defined according to a modified version of the 2011 Musculoskeletal Infection Society (MSIS) definition that was used clinically during the study period. Patients treated with a clindamycin-based therapy (used when first-line drugs were unsuitable) were included. Data were extracted manually from electronic records. Cure required no clinical or microbiological signs of recurrence of the same bacteria after 24 months of follow-up. Factors affecting treatment outcome were assessed using logistic regression analyses.

**Results:**

The two-year treatment success after clindamycin monotherapy (*n* = 46) and combination therapy (*n* = 62) at a typical dose of 300 mg three times daily was 82% (95% confidence interval [CI] 75–90%, *n* = 89/108). Two-year clindamycin treatment success following DAIR (*n* = 46), one stage exchange (*n* = 23) and two stage exchange (*n* = 32) were 72% (95% CI 58–85%), 91% (95% CI 79–100) and 94% (95% CI 85–100), respectively. Overall treatment success was 80% (95% CI 67–93%, *n* = 40) for patients receiving clindamycin/rifampicin. *Staphylococcus aureus* was independently associated with lower treatment success in a multivariable logistic regression OR 0.17 CI (0.05–0.64, *p* = 0.008). Side-effects were more frequent in patients receiving clindamycin + rifampicin (39%) compared to clindamycin monotherapy (10%) *p* = 0.002).

**Conclusions:**

Clindamycin (300 mg three times daily), administered as monotherapy or in combination with other antibiotics including rifampicin, appeared to be an effective and well-tolerated treatment for PJI. However, these regimens may be less effective in infections caused by *S. aureus*.

## Introduction

Prosthetic joint infections (PJI) are debilitating, costly and affect up to two per cent of all patients with prosthetic devices. Up to 80% of infections in hip and knee prosthesis are caused by Gram-positive bacteria [1]. Early PJI are typically treated with Debridement, Antibiotics, and Implant Retention (DAIR) [2]. After DAIR, first-line oral antibiotic treatment for infections caused by *Staphylococcus* species is rifampicin combined with ciprofloxacin or levofloxacin if the bacteria are susceptible and there are no contra indications [3]. Chronic PJI are typically treated by exchanging the prosthesis in one or two stages, followed by antibiotics [4].

Clindamycin is a lincosamide antibiotic with 87% bioavailability, that exhibits a bacteriostatic effect at standard dosing [5]. Clindamycin has excellent penetration to synovial fluid and bone [6]. It is active against many Gram-positive bacteria associated with PJI such as *Staphylococcus aureus*, *Streptococcus* spp., many strains of coagulase-negative *staphylococcus*, (CoNS) and > 90% of *Cutibacterium acnes*. Clindamycin is thus an attractive alternative when intolerance or resistance necessitates alternatives.

Combination treatment with rifampicin decreased the trough concentration of intravenously (IV) administered clindamycin by approximately 75% [7]. Similarly, oral co-administration of clindamycin and rifampicin reduced plasma trough and peak concentrations to 17%, and 34%, respectively, compared to concentrations obtained when clindamycin was given with levofloxacin [8]. Furthermore, co-administration of oral clindamycin and rifampicin resulted in a twelve-fold reduction of clindamycin AUC_0−8 h_, compared with oral clindamycin monotherapy in patients with bone and joint infections. It has consequently been suggested that only intravenous preparations of clindamycin should be used in combination with rifampicin in infections considered difficult to treat [9].

Data on the effectiveness and tolerability of clindamycin, either as monotherapy or in combination with rifampicin, for the treatment of PJI are scarce; only four small studies (6–36 patients) have reported success rates of 65–100% against *Staphylococcus* spp [10–13]. Nonetheless, clindamycin at a lower dose than generally recommended for PJI (300 mg three times a day) has been considered a well-tolerated and effective alternative for treatment of PJIs at our institution. This study aimed to describe the real-world clinical use of clindamycin, as mono or combination therapy, and to evaluate the effectiveness and tolerability of these regimens in patients with PJI.

## Methods

### Study design, setting and population

This was a retrospective single-centre study of patients treated for PJI, at Danderyd hospital, a 500-bed secondary hospital and regional referral centre for PJI in Stockholm, Sweden. The study was reported according to STROBE criteria [14]. Patients were identified from the outpatient register of the joint Orthopaedic and Infectious Disease outpatient clinic between January 1st, 2010, and December 31st, 2022. Additional patients were identified by searching orthopaedic department records for patients with any of the following ICD-10 diagnoses codes T845B, T845G, T845F, T840B, T840G and T840F. This ensured that all patients managed for PJI at the department of Orthopaedics were included minimizing any selection bias.

### Inclusion and exclusion criteria

Inclusion criteria were treatment of a PJI with clindamycin alone or in combination with another oral antibiotic.

Exclusion criteria were (A) Not fulfilling the criteria of PJI. (B) Having any part of the treatment (surgery, antibiotic treatment or follow up) done at another hospital. (C) Non-curative approach, i.e. patients that from the beginning were considered to have an infection needing lifelong antibiotic treatment.

### Definitions and variables

Many of the criteria (such as histology, alpha-defensin test and sonication) that are internationally used to define a PJI in the latest definition (EBJIS 2021) were not regularly used at our institution at the time of the study [15]. PJI was therefore defined according to the two major criteria of the Musculoskeletal Infection Society (MSIS) as follows; presence of a sinus tract communicating with the joint or growth of at least two phenotypically identical bacteria in intraoperative cultures [16]. For *S. aureus*, growth in at least one intraoperative culture was considered significant. Patients who received antibiotics prior to surgery were considered to have a PJI if at least one intraoperative 16S rDNA sequencing, from tissue or sonicated material, was positive, or if a preoperative arthrocentesis culture was positive.

Polymicrobial infections were accepted if at least one of the bacteria was sensitive to clindamycin. Polymicrobial infection was defined as identification of ≥ 2 bacteria fulfilling the criteria mentioned above.

PJI were categorized according to Tsukayama, as early postoperative infections, defined as infections that occur < 4 weeks after surgery, acute hematogenous infections defined as an infection with acute onset of symptoms occurring after a previously symptom-free postoperative period, or as late chronic infections defined as symptoms of infection for more than three weeks that occur after the first four postoperative weeks [17].

Antimicrobial susceptibility testing was done according to EUCAST Disk Diffusion, using disks from Thermofisher (Basingstoke, UK), at the laboratory of Clinical Microbiology, Karolinska hospital, Solna, Sweden.

The recommendation for intraoperative culture sampling at our institution during the time was ≥ 5 cultures taken with separate instruments. 16S rDNA sequencing was only recommended when a patient already was receiving antibiotic treatment. Typically, only one sample was taken for 16S rDNA sequencing. Sonication was performed at the discretion of the physician, and the result of the culture was reported without quantification.

Variables collected included demographics, comorbidities, diagnostics, microbiologic findings, surgical approach, antimicrobial therapy, and clinical outcome at the end of treatment and after one and two years. The following demographic variables have been defined accordingly:

- Healthy as no documented chronic illnesses.
- Obesity as BMI > 30.
- Immunosuppression as ongoing cytostatic, Disease Modifying Antirheumatic Drugs, or corticosteroids equivalent to ≥ 15 mg or prednisone daily.
- Malignancy as active solid tumour or haematological malignancy.

### Biases

A standardized data extraction form was used to ensure consistent data collection. Moreover, data was extracted before this specific hypothesis was generated meaning that data abstractors were essentially blinded to the study hypothesis and exposure status thereby reducing information bias. To minimise measurement bias functional outcomes were avoided and for example physical status was assessed using the American Society of Anesthesiologists (ASA) classification given preoperatively by an anaesthetist instead of trying to assess clinical frailty from patient notes. To minimise selection-bias all available patients were included as discussed above. Potential confounders were addressed by multivariate regression analyses.

### Outcome

The primary outcome was cure, defined as no clinical or microbiological signs of recurrence of the same bacteria within two years. Treatment failure was defined as any reoperation of the affected joint for proven or suspected infection, death attributed to infection, or the decision to extend the antibiotic treatment to lifelong treatment. Patients who were lost to follow up, died of causes not related to PJI or developed a new infection with new bacteria in the same joint, during the observational period, were censored and thus excluded from the outcome analysis.

Secondary outcomes included frequency of adverse events, and cure stratified by bacterial species and types of surgery.

### Data source

Data were extracted manually from electronic medical records (TakeCare, CompuGroup Medical Sweden AB) and registered in a REDCap™ database.

### Antimicrobial treatment

Standard empiric post operative IV therapy during the study period was cefotaxime plus vancomycin. When causative bacteria were identified directed antibiotic therapy was given. The duration of IV antibiotics was decided by the attending physician. Total antibiotic duration was recommended for twelve weeks after DAIR. After one-stage exchange arthroplasty, antibiotic therapy lasted 6 or 12 weeks. Antibiotic treatment was typically given for six weeks, followed by two weeks without antibiotics after the first step of a two-stage exchange arthroplasty. Antibiotic treatment should be withheld unless intraoperative cultures are positive after the second step.

The choice of oral antibiotic therapy followed the Swedish guidelines during the study period [18]. First-line treatment was a rifampicin-based therapy with quinolone as companion drug for *Staphylococcus* spp. after DAIR or one-stage exchange operations. If not applicable our second choice was clindamycin alone or in combination with rifampicin. For PJI caused by *Streptococcus* spp. or *C. acnes*, amoxicillin monotherapy was first choice, and clindamycin monotherapy was second choice antibiotic therapy, irrespective of type of operation. After the first step of a two-stage exchange operation the recommendation was to not give an antibiotic with biofilm-activity regardless of the bacteria, hence a beta lactam or clindamycin were the first choice for *Streptococcus* spp. *C. acnes* and *Staphylococcus* spp.

The dosing regimen of oral antibiotics were; clindamycin 300 mg or 450 mg three times a day, and rifampicin 600 mg once daily or 450 mg twice a day.

### Follow up

Patients were monitored at an outpatient clinic staffed by an Orthopaedic Surgeon and an Infectious Diseases specialist during antibiotic treatment. Patients were typically seen 2–3 times post discharge during antibiotic treatment with a final visit 2–3 weeks after the end of antibiotic treatment. Two-year outcomes were based on data extracted from electronic medical records.

### Statistics

Continuous variables are presented as medians with interquartile range (IQR) and categorical variables as numbers and percentages. When applicable 95% confidence intervals (CI) are presented. The CI was truncated at 100% if the sample was small and the confidence interval for the estimated proportion exceeded 100%. All available patients from when the current electronic medical record system was introduced were included thus determining study size. Univariate and multivariable binomial logistic regression models identified factors associated with treatment success at the 24-month follow-up, where positive associations indicated success. Variables with a sample of < 10 were not included due to low numbers. Variables with a p-value <0.1 in the univariate analysis were included in the multivariate analysis. The exposure variable was clindamycin treatment. Risk factors such as age, comorbidities type of surgery, and bacterial species were assessed as independent predictors of treatment outcome. A p-value < 0.05 was considered significant. Analyses were performed using Stata version 19 SE StataCorp. 2025.

## Results

**Fig. 1:**
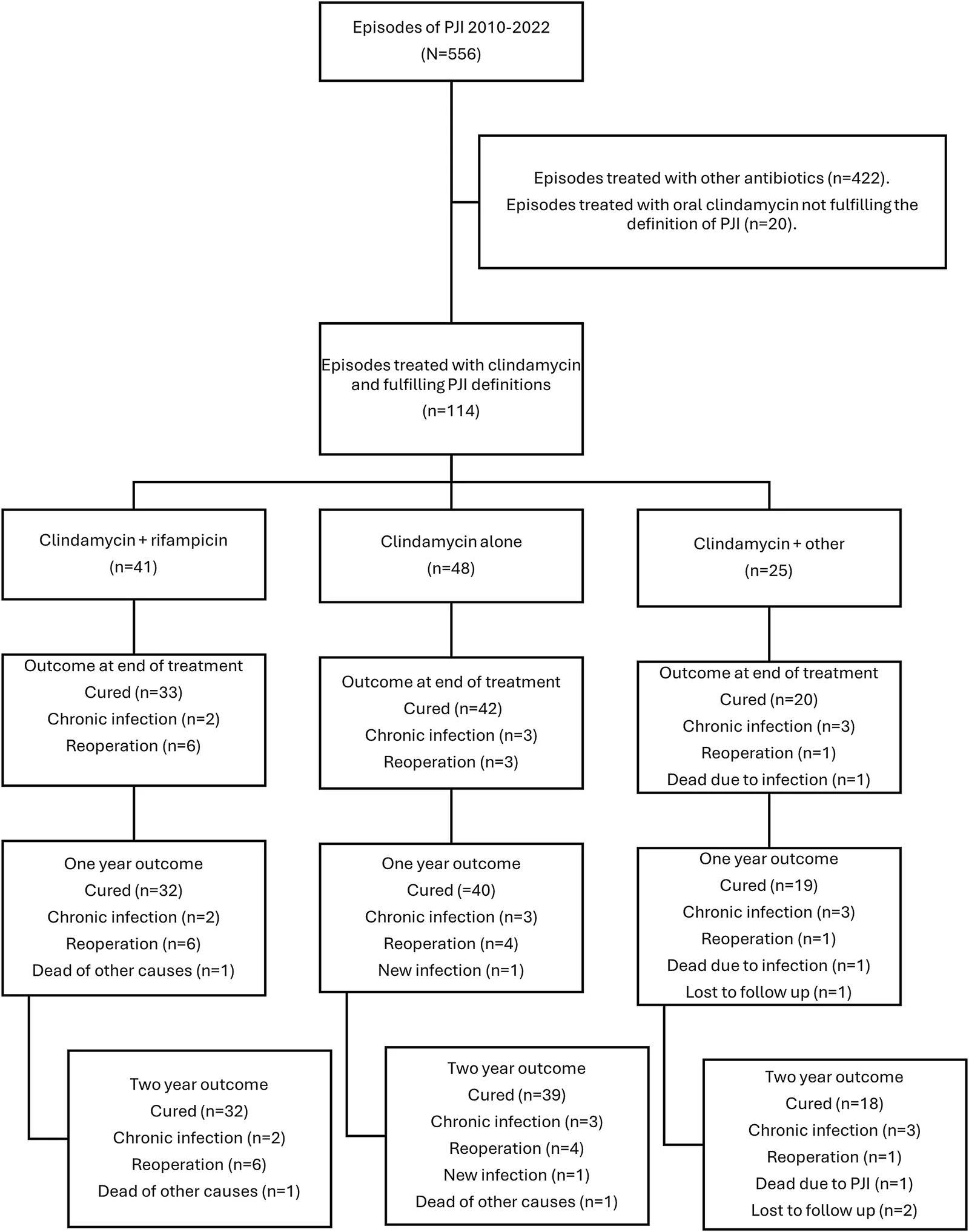
Flowchart showing patients and outcomes at the end of treatment and after one and two years

There were 556 episodes of PJI between 2010 and 2022. Oral clindamycin alone or in combination with another antibiotic was the treatment given for 134 episodes. Twenty patients were excluded from the analysis for not fulfilling the definition of PJI, as shown in Fig. 1. PJI diagnoses were based on ≥ 2 positive intraoperative tissue cultures in 96/114 cases (84%), cultures from preoperative arthrocentesis in 10/114 (9%), cultures from sonicated material in 2/114 (2%), and positive 16S rDNA sequencing in 2/114 (2%). Four (4%) cases were considered culture negative. Two of them had a single positive tissue culture during antibiotic treatment, one had a sinus tract communicating with the prosthesis, and one had visible pus during operation and a positive culture from sonicated material.

The demographics of the cohort are presented in Table 1. The median age of the cohort was 72 years (64–79). The majority were men (60%). The median ASA class was 2. 27% of patients did not have any comorbidities. The case mix was somewhat different between the groups with for example DAIR being the most common intervention in the clindamycin + rifampicin (63%) and clindamycin + other co-drug (56%) groups whilst clindamycin monotherapy was most common following two stage surgery (48%).

**Table 1:** Baseline demographics of patients treated with clindamycin for prosthetic joint infections

	All clindamycin	Clindamycin alone	Clindamycin + rifampicin	Clindamycin+ codrug^§^
**Number of patients**	114	48	41	25
Men	68 (60)	26 (54)	29 (71)	13 (52)
Age, years (IQR)	72 (64–79)	70 (61–77)	73 (68–80)	72 (64–79)
Weight, Kg (IQR)	83 (72–96)	84 (76–97)	87 (77–100)	78 (68–89)
BMI (IQR)	27 (24–30)	28 (26–30)	28 (25–31)	25 (23–28)
**Comorbidities**				
ASA class* 1	9 (8)	5 (11)	3 (8)	1 (4)
ASA class 2	46 (43)	24 (55)	16 (42)	6 (25)
ASA class 3	48 (45)	14 (32)	17 (45)	17 (71)
ASA class 4	3 (3)	1 (2)	2(5)	0 (0)
Healthy	30 (26)	12 (25)	12 (29)	6 (24)
Hypertension	61 (53)	26 (54)	20 (49)	15 (60)
Heart failure	11 (10)	2 (4)	5(12)	3 (12)
Diabetes	15 (13)	5 (10)	6 (15)	4 (16)
Renal failure	2 (2)	0 (0)	0 (0)	2 (8)
Immuno suppression	4 (4)	0 (0)	3 (7)	1 (4)
Dementia	4 (4)	3 (6)	0 (0)	1 (4)
Rheumatic disorder	9 (8)	4 (8)	2 (5)	3 (12)
Malignancy	8(7)	2 (4)	4 (10)	2 (8)
Smoking	7 (6)	0 (0)	3 (7)	4 (16)
Alcohol dependency	7 (6)	0 (0)	4 (10)	3 (12)
Obesity	18 (16)	7 (15)	8 (20)	3 (12)
**Infection site**				
Shoulder	9 (8)	7 (15)	1 (2)	1 (4)
Hip	74 (65)	34 (71)	25 (61)	15 (60)
Knee	31 (27)	7 (15)	15 (37)	9 (36)
**Type of infection**				
Acute postoperative	40 (35)	11 (23)	19 (46)	10 (40)
Acute haematogenous	25 (22)	9 (19)	10 (24)	6 (24)
Chronic	49 (43)	28 (58)	12 (29)	9 (36)
**Type of operation**				
DAIR^†^	49 (43)	9 (19)	26 (63)	14 (56)
One stage	23 (20)	11 (23)	10 (24)	2 (8)
Two stage	33 (29)	23 (48)	3 (7)	7 (28)
Girdlestone	5 (4)	4 (8)	0 (0)	1 (4)
Antibiotics only	4 (4)	1 (2)	2 (5)	1 (4)
**Effective initial IV treatment** ^α^	99/114 (87)	41/48 (85)	37/41 (90)	21/25 (84)
**Treatment duration**				
Intravenous (days)	8 (6–14)	8 (4–11)	8 (6–13)	16 (8–21)
Oral (days)	81 (48–89)	70 (44–84)	86 (69–101)	80 (62–95)
**Causative bacteria**				
*S. aureus*	24 (21)	5 (10)	10 (24)	9 (36)
CoNS^‡^	25 (22)	8 (17)	14 (34)	3 (12)
Polymicrobial Staphylococcal dominant infection	14 (12)	5 (10)	5 (12)	4 (16)
*Streptococcus* spp.	12 (10)	6 (12)	3 (7)	3 (12)
*C. acnes*	22 (19)	17 (35)	4 (10)	1 (4)
Polymicrobial infection	9 (8)	3 (6)	2 (5)	4 (16)
Culture negative	4 (4)	2 (4)	2 (5)	0 (0)
Other bacteria	4 (4)	2 (4)	1 (2)	1 (4)

Data are presented as number with percentages in brackets (%) or medians with interquartile ranges in brackets. *ASA American Society of Anaesthesiologists physical status classification. ^†^DAIR Debridement, Antibiotics and Implant Retention. ^‡^CoNS Coagulase negative *Staphylococcus*. ^§^Most common co-drugs were ciprofloxacin (*n* = 9) and fucidic acid (*n* = 8). ^α^ Causative bacteria were susceptible to administered intravenous antibiotics. Polymicrobial Staphylococcal dominant infection implies that at least one strain of *Staphylococcus* spp. was predominant. Polymicrobial infection implies that another specie than *Staphylococcus* spp. was predominant. Data on comorbidities are not available for all patients

### Postoperative intravenous antibiotic treatment

Median IV antibiotic treatment duration was 8 (IQR 6–14) days. The most frequently used IV antibiotics were vancomycin (45%) and cefotaxime (39%). Twenty-eight per cent of the patients received those two antibiotics in combination. Clindamycin and cloxacillin were used as initial therapy in 24 and 22%, respectively. Empiric therapy covered the causative bacteria in 95/110 (86%) of the patients in whom bacteria were identified. One hundred and five culture-positive patients were evaluable after 24 months. Treatment was successful in 74/90 (82%) of the patients that received effective IV empiric therapy compared to 12/15 (80%) of patients who received incorrect treatment (*p* = 0.73).

### Oral antibiotic treatments

Clindamycin, alone or with a companion drug, was given as oral follow up treatment for a median of 81 (48–89) days. Clindamycin doses were either 300 mg (104/114) or 450 mg (10/114) three times daily. Cure occurred in 82/99 (83%) and 7/9 (78%) of patients treated with 300 mg and 450 mg of clindamycin three times daily, respectively (*p* = 0.66). Rifampicin doses were 600 mg once daily (35/41) or 450 mg twice daily (6/41). There was no difference in two-year outcome, 27/34 (79%) vs. 5/6 (83%), (*p* = 0.82).

### Effectiveness of clindamycin backbone therapy for treatment of prosthetic joint infections

Two years after surgery 108/114 patients were evaluable. Among the six patients that were excluded, two were lost to follow up, two died of other causes, one had a new infection with new bacteria, and in one case the decision to give lifelong antibiotic treatment was taken during the operation. Outcomes at the end of treatment, after one and two years in each treatment group are presented in Fig. 1. Cure rates at the end of antibiotic treatment, and after one and two years for the clindamycin backbone therapy group were 95/113 (84% [95% CI 77–91%]), 91/110 (83% [95% CI 76–90%]) and 89/108 (82% [95% CI 75–90%]), respectively. Data on two-year outcomes stratified for treatment group, surgical procedure and bacteria are presented in Table 2. The overall two-year success rates following DAIR (*n* = 46), one stage exchange (*n* = 23) and two stage exchange (*n* = 32) after treatment with clindamycin mono or combination therapy were 72% (95% CI 58–85%), 91% (95% CI 79–100%) and 94% (95% CI 85–100%), respectively.

**Table 2:** Treatment success two years after surgery for prosthetic joint infection stratified by oral antibiotic therapy and type of surgery

	All Clindamycin	Clindamycin Only	Clindamycin + Rifampicin	Clindamycin + other
Number of patients	108	46	40	22
Total treatment success	89 (82)	39 (85)	32 (80)	18 (82)
Treatment failure	19 (18)	7 (15)	8 (20)	4 (18)
Censored	6	2	1	3
**Treatment success after DAIR**				
All	33/46 (72)	6/9 (67)	17/25 (68)	10/12 (83)
*Staphylococcus* spp.	16/25 (64)	0/1 (0)	10/16 (62)	6/8 (75)
*S. aureus*	6/14 (43)	0/1 (0)	3/8 (38)	3/5 (60)
CoNS	6/7 (86)	ND	5/6 (83)	1/1 (100)
Polymicrobial staphylococcal dominant infection	4/4 (100)	ND	2/2 (100)	2/2 (100)
*C. acnes*	5/6 (83)	1/2 (50)	4/4 (100)	ND
*Streptococcus* spp.	7/8 (88)	4/4 (100)	1/2 (50)	2/2 (100)
Polymicrobial infection	3/4 (75)	0/1 (0)	1/1 (100)	2/2 (100)
Other bacteria and culture negative^1^	2/3 (67)	1/1 (100)	1/2 (50)	ND
Oral treatment duration (days)	84 (73–93)	84 (81–95)^2^	88 (75–111)	81 (67–89)
**Treatment success after one stage surgery**				
All	21/23 (91)	10/11 (91)	10/10 (100)	1/2 (50)
*Staphylococcus* spp.	12/13 (92)	2/2 (100)	9/9 (100)	1/2 (50)
*S.aureus*	1/2 (50)	ND	ND	1/2 (50)
CoNS	8/8 (100)	2/2 (100)	6/6 (100)	ND
Polymicrobial staphylococcal dominant infection	3/3 (100)	ND	3/3 (100)	ND
*C. acnes*	6/6 (100)	6/6 (100)	ND	ND
*Streptococcus* spp.	1/1 (100)	1/1 (100)	ND	ND
Polymicrobial	1/1 (100)	1/1 (100)	ND	ND
Other bacteria and culture negative^1^	1/2 (50)	0/1 (0)	1/1 (100)	ND
Oral treatment duration (days)	84 (81–93)	84 (80–93)	87 (84–101)	-
**Treatment success after two-stage surgery**				
All	30/32 (94)	21/22 (95)	3/3 (100)	6/7 (86)
*Staphylococcus* spp.	15/16 (94)	11/12 (92)	1/1 (100)	3/3 (100)
*S. aureus*	4/4 (100)	3/3 (100)	ND	1/1 (100)
CoNS	6/6 (100)	4/4 (100)	1/1 (100)	1/1 (100)
Polymicrobial staphylococcal dominant infection	5/6 (83)	4/5 (80)	ND	1/1 (100)
*C. acnes*	8/8 (100)	7/7 (100)	ND	1/1 (100)
Streptococcus spp.	3/3 (100)	1/1 (100)	1/1 (100)	1/1 (100)
Polymicrobial inf	3/3 (100)	1/1 (100)	1/1 (100)	1/1 (100)
Other bacteria and culture negative^1^	1/2 (50)	1/1 (100)	ND	0/1 (0)
Oral treatment duration (days)^3^	47 (35–68) +	47 (40–59) +	-	61 (35–112) +
	15 (0–31)	17 (0–42)		0 (0–31)
**Treatment success after Girdlestone or antibiotics alone**	5/7 (71)	2/4 (50)	2/2 (100)	1/1 (100)
All staphylococcal spp.	3/4 (75)	1/2 (50)	2/2 (100)	ND

Data are presented as number with percentages in brackets (%) or medians with interquartile ranges in brackets. Polymicrobial Staphylococcal dominant infection implies that at least one strain of *Staphylococcus* spp. was predominant. Polymicrobial infection implies that another specie than *Staphylococcus* spp. was predominant. ^1^Other bacteria were *Bacteroides* spp. (*n* = 3), *Bacillus* spp. (*n* = 1), and culture negative (*n* = 4). ^2^Due to low numbers mean days are given instead of median. ^3^ Number of days with antibiotic treatment after stage one followed by number of days after stage two

Two-year success rates per joint were as follows; shoulders 9/9 (100%), hips 57/70 (81% [95% CI 72–91]), and knees 23/29 (79% [95% CI 64–95]) and were not significantly different (*p* = 0.45).

Two-year success rates per bacterial species were as follows:

- *C. acnes* − 20/21 (95% [95% CI 85–100%])
- *Streptococcus* spp. − 11/12 (92% [95% CI 73–100%]), (9 beta-haemolytic *streptococcus* and three alpha-haemolytic *streptococcus*)
- *Staphylococcus* spp. − 34/45 (76% [95% CI 63–88%])
- monomicrobial plus polymicrobial infections with staphylococcal dominance − 46/58 (80% [95% CI 69–90%]).

### Factors associated with treatment outcome two years post-surgery in patients treated with clindamycin

One hundred and eight clindamycin-treated patients were included in uni- and multivariable analysis as presented in Table 3. The only factor that was significantly associated with two-year treatment outcome in the multivariable analysis was infection with *S. aureus* with an OR of 0.17 (95% CI, 0.05–0.64, *p* = 0.008).

Outcomes for patients treated with the combination of clindamycin and rifampicin 32/40 (80%), clindamycin alone 39/46 (85%) and clindamycin together with another antibiotic 18/22 (82%) were not significantly different (*p* = 0.80).

### Patients with treatment failure in the clindamycin + rifampicin group

Of the eight patients in the clindamycin/rifampicin group that were not cured, six were reoperated. Three had negative intraoperative cultures at the time of reoperation. Three were still positive for the same bacteria of which one had a *S. aureus* infection and needed a reoperation after only one week of clindamycin and rifampicin therapy. The other two were caused by beta-haemolytic streptococcus and *Bacteroides fragilis*, respectively and relapsed after completing three months of treatment. None of the isolates were resistant to rifampicin.

**Table 3:** Uni- and multivariate analyses showing odds ratio for treatment success in patients treated with clindamycin-based therapy for prosthetic joint infections at Danderyd hospital 2010–2022

*N* = 108	Univariate analyses			Multivariate analyses		
Variable	OR	95% CI	P-value	OR	95% CI	P-value
Age	0.96	0.91–1.01	0.08	1.00	0.95–1.08	0.87
Sex male: female	3.37	1.20–9.45	0.02	3.57	0.88–14.56	0.08
BMI	0.95	0.85–1.07	0.39			
Healthy (*n* = 30)	4.1	0.89–19.0	0.07	3.92	0.68–22.55	0.13
Hypertension (*n* = 61)	0.35	0.12–1.05	0.06			
Heart failure (*n* = 10)	0.45	0.10–1.95	0.29			
Diabetes (*n* = 15)	0.67	0.17–2.73	0.58			
Obesity (*n* = 18)	0.7	0.20–2.42	0.57			
Asa 1–2 versus > 2	2.21	0.77–6.35	0.14			
**Infection site**						
Hip versus not hip (*n* = 70)	0.82	0.28–2.37	0.72			
Knee versus not knee (*n* = 29)	0.75	0.25–2.21	0.61			
Shoulder	ND	ND	ND			
**Type of infection**						
Acute post operative or not (*n* = 36)	1.49	0.49–4.54	0.48			
Acute haematogenous or not (*n* = 24)	0.22	0.08–0.65	0.006	0.27	0.07–1.04	0.06
Chronic postoperative or not (*n* = 48)	2.62	0.87–7.88	0.09			
**Type of surgery**						
Exchange vs. DAIR operations (*n* = 55/46)	5.02	1.51-16:7	0.009	2.56	0.62–10.65	0.20
**Causative bacteria**						
All *staphylococcus* spp (*n* = 58)	0.62	0.22–1.73	0.36			
*Staphylococcus aureus* (*n* = 21)	0.17	0.06–0.51	0.002	0.17	0.05–0.64	0.008
Coagulase-negative *staphylococcus* (*n* = 24)	2.79	0.60–13.0	0.19			
Polymicrobial infection with Staphylococcal predominance (*n* = 13)	2.81	0.34-23	0.34			
Polymicrobial infection (*n* = 9)	1.78	0.21–15.1	0.60			
*Cutibacterium acnes* (*n* = 21)	5.21	0.66–41.5	0.12			
*Streptococcus* spp (*n* = 12)	2.54	0.31–20.9	0.39			

### Side effects

Side effects were reported by 25 patients, see Table 4. Five patients stopped their treatment due to side effects. The most common side effect was itching/rash (*n* = 9) followed by nausea/vomiting (*n* = 5) and diarrhoea (*n* = 5). Side effects were reported by 16/41 (39%) of patients taking clindamycin plus rifampicin and 5/48 (10%) of patients taking clindamycin alone (*p* = 0.002). Having a rash or itching was more common in the group receiving clindamycin plus rifampicin group. (*p* = 0.02). One patient treated with rifampicin was hospitalised for suicidality and thus fulfilled the definition of a serious adverse event (SAE) [19]. Two patients had elevated liver enzymes. In one case cotreatment was with rifampicin and ASAT/ALAT was tripled compared to baseline. The other case was most probably due to overuse of alcohol.

**Table 4:** Reported side effects in patients treated for prosthetic joint infection at Danderyd Hospital between 2010 and 2022

	All	Clindamycin + rifampicin	Clindamycin alone	Clindamycin + other
Number of patients	114	41	48	25
Any side-effect	25 (22%)	16^†^ (39%)	5^†^ (10%)	4 (16%)
Antibiotics stopped due to side effects	5 (5%)	3(7%)	1 (2%)	1 (4%)
Rash/itch	10 (9%)	8^‡^ (20%)	2^‡^ (4%)	0 (0%)
Nausea/vomiting	5 (4%)	3 (7%)	1 (2%)	1 (4%)
Diarrhea	5 (4%)	3 (7%)	2 (4%)	0 (0%)
*Clostridioides difficile* enteritis	0 (0%)	0 (0%)	0 (0%)	0 (0%)
Weight loss of > 10%	1 (1%)	1 (2%)	0 (0%)	0 (0%)
Affected blood chemistry	3 (3%)	1 (2%)	0 (0%)	2 (8%)
Unspecified*	1 (1%)	1 (2%)	0 (0%)	0(0%)

^*^Attempted suicide

^†^*p* = 0,002

^‡^*p* = 0,02

## Discussion

Rifampicin together with a quinolone have been the backbone antibiotic therapy for PJI caused by *Staphylococcus* spp. for the past twenty-five years [20]. Resistance among *S. aureus* to rifampicin and quinolones is rare but interactions with other drugs are common [21]. Rifampicin-based therapy is consequently often unsuitable in an elderly population due to polypharmacy and alternative antibiotics are needed. Clindamycin is a potential alternative that has been used at our site when first line treatment was unsuitable, but which is currently not recommended by the Infectious Diseases Society of America for the treatment of PJI following DAIR [3]. The aim of this study was thus to assess the effectiveness of clindamycin given at a lower dose than normally recommended, alone or in combination with another antibiotic for treatment of PJI at a single centre over a 13-year period.

Finding an overall two-year cure rate of 82% for the whole clindamycin cohort – comprising 72% for DAIR and 93% for exchange operations - is encouraging. Infections caused by *C. acnes* and *Streptococcus spp.* had success rates of 83–100%, regardless of surgical procedure. The cure rate was lower at 62% (16/25) for PJI caused by *Staphylococcus spp.* in the DAIR group, driven largely by a low 43% (6/14) success rate for *S. aureus*. However, this Staphylococcal DAIR outcome aligns with both a meta-analysis of DAIR and a recent multicentre study of 669 patients where the one-year cure rates were 68% with rifampicin-based therapy and 46% without rifampicin [22, 23]. The two-year success rate for exchange operations was similar regardless of treatment regimen, and bacterial species, but numbers in some groups are small, making meaningful comparisons difficult to make. However, a 2021 meta-analysis corroborates the high exchange operation success rates, showing similar outcomes for one stage (87%) and two stage (83%) revisions of infected knee arthroplasties [24]. Thus clindamycin, alone or in combination with another antibiotic, including rifampicin, appeared to be an effective treatment against low-virulent bacteria such as *C. acnes*, CoNS spp. and the more virulent beta-haemolytic *streptococcus* spp. but its effectiveness appeared to be limited against *S. aureus* as discussed in more detail below.

Even though the two-year cure rate for all staphylococcal infections treated with clindamycin was 80%, only 57% (12/21) of infections caused by *S. aureus* were cured. In line with this, *S. aureus* was associated with an adjusted OR for treatment success of 0.17 (95% CI, 0.04–0.64, *p* = 0.008) in the multivariate analyses of all patients treated with clindamycin. Moreover, *S. aureus* is a known risk factor for treatment failure [25]. The two-year success rates of DAIR treated *S. aureus* PJIs were 3/8 (38%) following clindamycin/rifampicin and 3/5 (60%) following treatment with clindamycin and another antibiotic. For comparison, the success rate for infections caused by *S. aureus* following treatment with ciprofloxacin + rifampicin and DAIR at our site for the same study period was 73% (30/41) (unpublished data). The low success rate suggests that a clindamycin-based therapy may not be optimal. However, the number of patients was very small, intraoperative cultures amongst patients treated with clindamycin + rifampicin were negative in 3/6 patients when reoperated for treatment failure and there will be biases in the choice of treatment making it difficult to interpret the results.

Treatment success in PJI caused by CoNS and polymicrobial infection with predominance of *Staphylococcus* spp. treated with DAIR had a better result, 10/11 (91%) were cured. Eight of the patients were treated with clindamycin + rifampicin. All patients (8/8), with a CoNS infection and treated with a one-stage exchange operation were cured. Six of them were treated with clindamycin and rifampicin in combination. Patients treated with a two-stage operation had a similar total success rate of 100% (6/6). In this group the majority were treated with clindamycin alone. The results indicate that in infections caused by CoNS, clindamycin together with another antibiotic, including rifampicin, could be an alternative.

*C. acnes* was the second most common bacteria causing PJI in this cohort. The 95% cure rate was similar to previous reports in which *C. acnes* were treated both with and without the addition of rifampicin to standard treatment that is most often either amoxicillin or clindamycin [26]. The relevance of *C. acnes* in PJI has been questioned since *C. acnes* sometimes occurs without signs of inflammation suggesting that it should be considered as a commensal and not a pathogen [27]. However, in 17/21 cases *C. acnes* grew in at least three different cultures, indicating at least an abundance of the bacteria. Moreover, only 4/21 infections were in shoulders, where *C. acnes* are more common and pre-surgery C-reactive protein (CRP) or erythrocyte sedimentation rate (ESR) levels were over 50 in 10/13 patients indicating a true infection.

Nine patients with beta-haemolytic streptococcus and three with alpha-haemolytic streptococcus were included in the cohort of which 8 were treated with DAIR. The cure rate was high in this group with 11/12 patients cured after 24 months. Our results are in line with the 88% one-year cure reported in a retrospective cohort with 81 patients treated for streptococcal PJI [28]. This indicates that PJI caused by *Streptococcus* spp. can be managed using standard antibiotic treatment durations as opposed to lifelong antibiotic suppression that has been suggested [29].

Rifampicin is a well-known inducer of the cytochrome P450 system and enhances the elimination of several antibiotics including clindamycin. Pharmacokinetic modelling of IV and oral clindamycin showed that rifampicin increased clindamycin clearance threefold and decreased the bioavailability of clindamycin from 56% to 11% and 4% when concomitantly administrated with 600 mg and 900 mg of rifampicin given twice a day respectively. Based on this data 1200 mg of clindamycin four times daily would be needed, to have an 81% chance of attaining concentration equal to the clinical breakpoint for *S. aureus* [30]. This suggests that it is highly unlikely that any of the patients in our cohort would have reached the target MIC or four times the MIC based on breakpoints from the European committee of antimicrobial susceptibility testing, (*Staphylococcus* spp. 0.25 mg/L, *Streptococcus* spp. 0,5 mg/L and *C. acnes* 0,25 mg/L) [31]. However, the 80% (95% CI 67–93%) effectiveness of clindamycin/rifampicin given to 40 patients (37 received clindamycin 300 mg three times a day), suggests that the clinical impact of rifampicin’s interaction with clindamycin is negligible at the doses used in this study. This could be due to a reduced induction of the P450 system with the lower rifampicin doses used in this cohort or that the effectiveness is primarily due to rifampicin alone, and that clindamycin´s role is merely to prevent emerging resistance to rifampicin or to surgery alone but it is beyond the scope of this article to determine this.

The data thus suggests that lower clindamycin concentrations induced by rifampicin do not have a clinical impact in PJI caused by Gram-positive bacteria other than possibly *S. aureus*. Moreover, treatment outcomes were similar in the four other cohorts found in literature that described the effectiveness of clindamycin (typically 600 mg three times daily) + rifampicin for treatment of staphylococcal bone and joint infections. At least 32/69 patients in those studies underwent DAIR and overall success rates were 86%, 65%, 70%, 100% respectively [10–13]. The data is limited by small numbers but are in line with different *S. aureus* and CoNS outcomes reported in a meta-analysis focusing on staphylococcal PJIs treated with DAIR [32].

Adverse events were almost four times more frequent in the group that received clindamycin/rifampicin compared to the patients that received clindamycin alone (*p* < 0.001) indicating that the side effects were often due to rifampicin rather than to clindamycin. In line with this, 25% of patients receiving ciprofloxacin/rifampicin for PJI at our site during the same study period reported adverse events (unpublished data). Adverse events resulting in treatment being stopped occurred in 5% of all patients which is similar to the 6% reported by a study on adverse events after PJI [33]. However, treatment appeared to be more often stopped (non-significant difference) in the clindamycin + rifampicin group compared to the clindamycin monotherapy group. Clindamycin monotherapy also seemed to be well tolerated when compared to a Swedish study on linezolid and PJI that reported adverse events in 39% of patients [34]. Moreover, the number of diarrhoeal episodes were few in all groups and no cases of *C. difficile* occurred, further indicating that clindamycin is a well-tolerated alternative for the treatment of PJI.

This study has several limitations. It is a retrospective cohort, numbers are small, there are no data on serum concentrations of clindamycin, there were no cases of MRSA infection, the cohort consists of both DAIR and exchange operations and includes different bacteria, of whom some are biofilm producers. Moreover, though the two-year outcome was similar in the clindamycin monotherapy, clindamycin + rifampicin and clindamycin + other codrug treatment groups, the case mix between the groups was different (indication bias) making comparisons difficult. For example, there was a higher proportion of chronic infections and consequently a higher proportion of exchange operations in the group treated with clindamycin monotherapy. Also, side-effects were not asked for in a standardized manner. Strengths include that it is one of the largest studies on PJIs treated with clindamycin, the data represents a real-world scenario with little loss to follow-up, and we can present reliable data on clinical outcome up to two years.

## Conclusion

In this study clindamycin (300 mg three times a day), given alone or in combination with another antibiotic, including rifampicin, appeared to be a well-tolerated and effective treatment against PJI caused by low-virulent bacteria such as *C. acnes*, CoNS spp. and the more virulent beta-haemolytic *streptococcus.* Infections caused by *S. aureus* had a worse outcome. Despite the weaknesses of this study, a low dose clindamycin-based therapy may be an alternative when standard treatment cannot be used, but further studies are needed.

## Data Availability

Limited datasets, without any personal or identifiable information, used and/or analysed during the current study are available from the corresponding author, upon reasonable request.

## Abbreviations

PJI: Prosthetic joint infection
DAIR: Debridement, antibiotics, implant retention
IV: Intravenously
AUC: Area under the curve
STROBE: Strengthening the reporting of observational studies in epidemiology
MSIS: Musculoskeletal Infection Society
EUCAST: European committee of antimicrobial susceptibility testing
ASA: American Society of Anesthesiologists
CI: Confidence interval
CoNS: Coagulase negative staphylococcus
T84.0B: Mechanical complication of internal joint prosthesis in the shoulder joint
T84.0G: Mechanical complication of internal joint prosthesis in the knee joint
T84.0F: Mechanical complication of internal joint prosthesis in the hip joint
T84.5B: Infection and inflammatory reaction due to internal joint prosthesis in the shoulder joint
T84.5G: Infection and inflammatory reaction due to internal joint prosthesis in the knee joint
T84.5F: Infection and inflammatory reaction due to internal joint prosthesis in the hip joint
